# Identification, Biological Function Profiling and Biosynthesis of Secondary Metabolites in Medicinal Orchids

**DOI:** 10.3390/metabo13070829

**Published:** 2023-07-07

**Authors:** Kunqian Li, Fengju Wu, Mengzhu Chen, Zhihao Xiao, Ya Xu, Mengwei Xu, Jingyi Liu, Delin Xu

**Affiliations:** 1 Department of Medical Instrumental Analysis, Zunyi Medical University, Zunyi 563099, China; mfkunqian@163.com (K.L.);; 2 Department of Cell Biology, Zunyi Medical University, Zunyi 563099, China

**Keywords:** medicinal orchid, metabolite biosynthesis, chemical identification, function profiling

## Abstract

The secondary metabolites present in medicinal orchids are diverse and possess a vast array of biological activities. They represent valuable raw materials for modern pharmaceuticals and clinical medicine and have tremendous potential for future development. A systematic collation of secondary metabolites’ composition and a summary of the biological activities of medicinal orchids represent a crucial step in unlocking the potential of these valuable resources in drug development. Furthermore, such information can provide essential guidance for comprehensively analyzing the pharmacological and therapeutic mechanisms of these valuable herbs in traditional Chinese herbal medicine. This review article presents an overview of the types and main biological functions of the secondary metabolites found in medicinal orchids, as well as the conventional synthesis methods for these compounds. Our aim is to provide a useful reference for future research and the drug development of secondary metabolic products of medicinal orchids.

## 1. Introduction

Orchids have a long history of medicinal use in China due to their cultivation for over 2000 years. The orchid family is diverse, and so far, over 28,000 species have been identified in 736 genera [1]. In the 2020 edition of the Chinese Pharmacopoeia, several species were recorded as Chinese herbal medicines (CHM). These include *Gastrodia elata*, *Bletilla striata*, *Dendrobium nobile*, *D. huoshanense*, *D. chrysotoxum*, *D. fimbriatum*, *D. officinale*, *Cremastra appendiculata*, *Dendrobium* spp., *Pleione bulbocodioides* and *P. yunnanensis*. In China, wild orchids have a long history as medicinal and ornamental plants. Due to their ability to treat diseases and enhance beauty and health, orchids have now become a new economic crop in agriculture. The development of orchids has resulted in several social benefits, particularly in promoting “green and natural” dietary options and daily essentials, including decoction pieces, oral liquids, beauty products, and skincare solutions. Moreover, the active elements identified in orchid plants can be leveraged to formulate new drugs. In this paper, ten genera of medicinal orchids are introduced, primarily focusing on *Dendrobium*. These genera include *Bletilla*, *Anoectochilus*, *Liparis* and several others. Research findings have shown that the types and quantities of the secondary metabolites present in CHM are known to play a significant role in their pharmacological effects and medicinal quality [2]. To effectively utilize medicinal orchid resources, it is particularly important to excavate their secondary metabolites.

In recent years, secondary metabolites have gained public attention due to their diverse biological activities and pharmacological effects, making them promising candidates for developing new drugs for stubborn diseases. The secondary metabolites found in medicinal orchids are small-molecule substances with various characteristics, including diversity, high specificity and rich biological functions. This makes them a potential source of highly specific active substances to treat stubborn diseases. However, research on medicinal orchids primarily focuses on species identification, classification, conservation and cultivation, as well as compound identification. More attention needs to be paid to the analysis and use of active substances derived from secondary metabolites, which are still in early stages. Despite their potential, medicinal orchids face natural limitations, such as their growth cycle, season, climate and natural accumulation of compounds, resulting in a situation similar to paclitaxel’s in which there is low market supply and high market demand. To address this, it is crucial to elucidate the active secondary metabolites and biosynthesis of medicinal orchid secondary metabolites leading towards resource development and utilization.

To promote the production of medicinal orchids and the development and utilization of secondary metabolites from their active secondary metabolites, this article provides a review of the separation and identification processes, biological functions and biosynthesis methods of the secondary metabolites of medicinal orchids. The objective is to offer useful references for future research on medicinal orchids and their secondary metabolites, which are crucial for clinical applications.

## 2. Identification of Secondary Metabolites

These compounds were first proposed by the German chemist Kossel in 1891, and they are small molecules synthesized by organisms in response to environmental stress and resistance. They serve as plant protectants that aid in disease resistance and defense against natural enemies [3,4]. The biological effects of secondary metabolites are the result of species evolution and adaptation to the environment. The secondary metabolites of medicinal orchids can be classified into three groups: terpenoids, phenols and nitrogen compounds, each of which contain tens of thousands of compounds. Furthermore, the production and distribution of these secondary metabolites are species specific and only occur in particular organs, tissues and developmental stages of the orchids [4]. For instance, *D. nobile* has the largest number of dendrobine types, whereas *D. chrysotoxum* has the highest content of moscatilin, with stem tissue being the primary medicinal component. The active substances in traditional Chinese medicine are the material basis for their pharmacological effects. Therefore, the identification and analysis of secondary metabolites are necessary prerequisites for the development and application of medicinal orchids’ active substances. In this review, 155 secondary metabolites from medicinal orchids were identified for the first time between 2018 and 2023, including alkaloids, phenanthrenes, bibenzyl, flavones and coumarins (refer to Table 1).

### 2.1. Alkaloids

**Figure 1:**
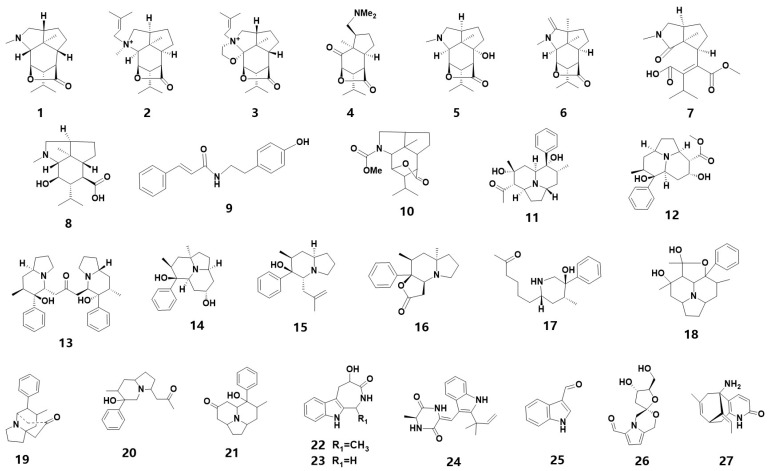
The chemical structure of alkaloids found in orchids.

Alkaloids are the most commonly found nitrogen-containing compounds in the secondary metabolites of medicinal orchids and an essential source of bioactive compounds. More than 50 species of orchids have yielded the discovery of over 140 alkaloids [5], which can be classified into various groups based on their structural characteristics, such as sesquiterpenes, indolizines, amides and indoles. In 2019, 52 alkaloid components were detected in 19 orchid species [6]. However, the alkaloid content in medicinal orchids is typically low, with only five out of thirty-five *Dendrobium* plants having an alkaloid content of over 0.1% [7]. This paper summarizes 27 newly identified alkaloids, whose chemistry structure are shown in Figure 1. It is worth noting that three new alkaloids were identified from *D. nobile*, including a new pair of amide tautomers [8]. Four new indolizine alkaloids were identified from *D. crepidatum* [9,10]. Furthermore, a pair of enantiomers and three new indolizine alkaloids have been discovered from *D. crepidatum* [11]. These twenty-seven alkaloids are listed in Table 1 and include eight sesquiterpene alkaloids (1–8), two amides (9–10), eleven indolizines (11–21), four indoles (22–25), a spiral-shaped alkaloid (26) and a Lycodine-type *Lycopodium* alkaloid (27).

### 2.2. Phenanthrenes

**Figure 2:**
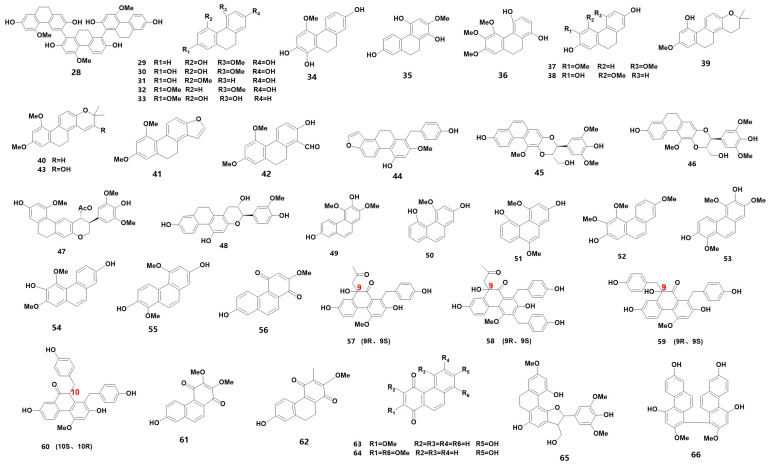
The chemical structure of phenanthrene found in orchids.

Phenanthrenes are a class of substances with three benzene rings as the parent ring and are commonly found in medicinal orchids. Based on their structural properties, they can be classified into simple phenanthrene, dihydrophenanthrene (DHP), phenanthraquinone (ketone), phenanthrene furan and phenanthrene dimer. Table 1 summarizes the 39 phenanthrenes that were identified from medicinal orchids between 2018 and 2023 (for their chemical structures, refer to Figure 2). Notably, a new DHP trimer was identified from *B. striata* [12], and silk grass was found to contain six new DHP [13]. Furthermore, monolithic orchids were discovered to have four optically rotating phenanthraquinones [14], and two new phenanthraquinones were found in *Dendrobium* flowers [15]. Additionally, three secondary metabolites were identified for the first time from plants in the genus *Bletilla* [12]. These thirty-nine phenanthrenes are listed in Table 1 and include one DHP dimer (28), twenty DHP (29–48), seven simple phenanthrenes (49–55) and nine phenanthraquinones (56–64), as well as furans (65) and biphenanthrene (66).

### 2.3. Bibenzylates

**Figure 3:**
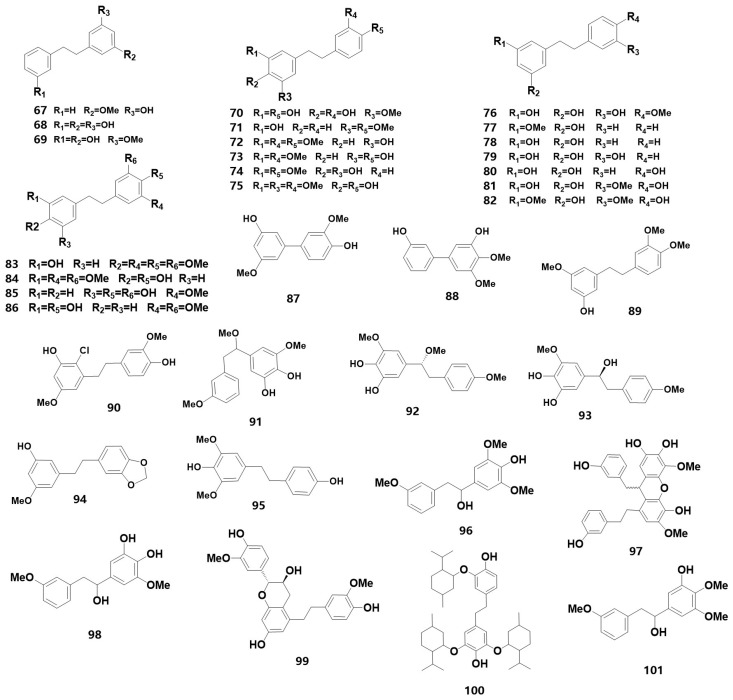
The chemical structure of bibenzyl substances found in orchids.

Bibenzyl is an essential precursor for synthesizing phenanthrene substances, with 1,2-diphenylethane as the parent ring. Among medicinal orchids, the bibenzyls of *Dendrobium* have been studied in depth. For instance, eight bibenzyl compounds were isolated from *D. officinale* leaves [16], and its stems contained 15 bibenzyl compounds, including dendrocandin X and 3,4′-dihydroxy-4,5-dimethoxybibenzyl, which were isolated from *D. officinale* for the first time [17]. Additionally, dendrocandin Y, a new bibenzyl derivative, was discovered from the stem extract [18]. Using UPLC-Q-TOF-MS technology, five bibenzyloids, including densiflorol A, aloifol I and isomoniliformin A, were identified in *D. pendulum* for the first time [19]. The discovery of new benzyl compounds in medicinal orchids serves as a research resource for developing their medicinal value. Table 1 (67–101) summarizes the information of 35 bibenzyls (for their chemical structures, refer to Figure 3). In Table 1, composition (67) was identified as new from *Bletilla*, whereas compositions (75, 83, 89) were determined to be new from *Dendrobium*. Three new substances (90, 98, 101) were found in *D. plicatile* [20], *D. hercoglossum* [21] and *D. hancockii* [22], respectively, within the genus.

### 2.4. Other Secondary Metabolites

**Figure 4:**
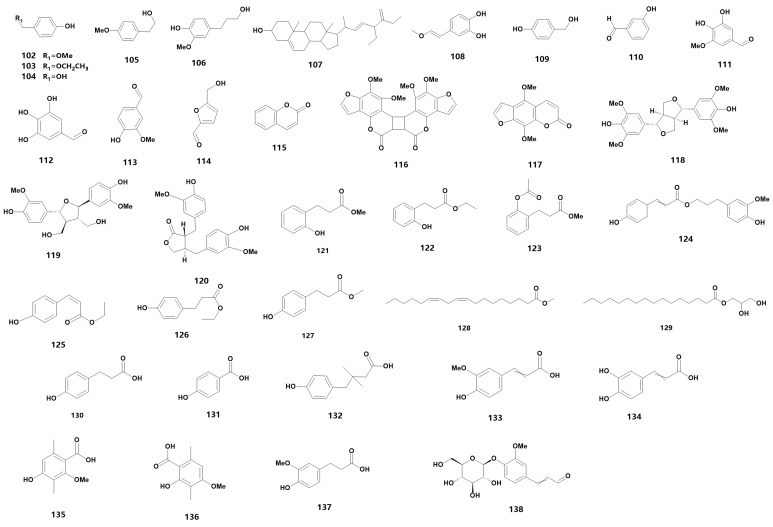

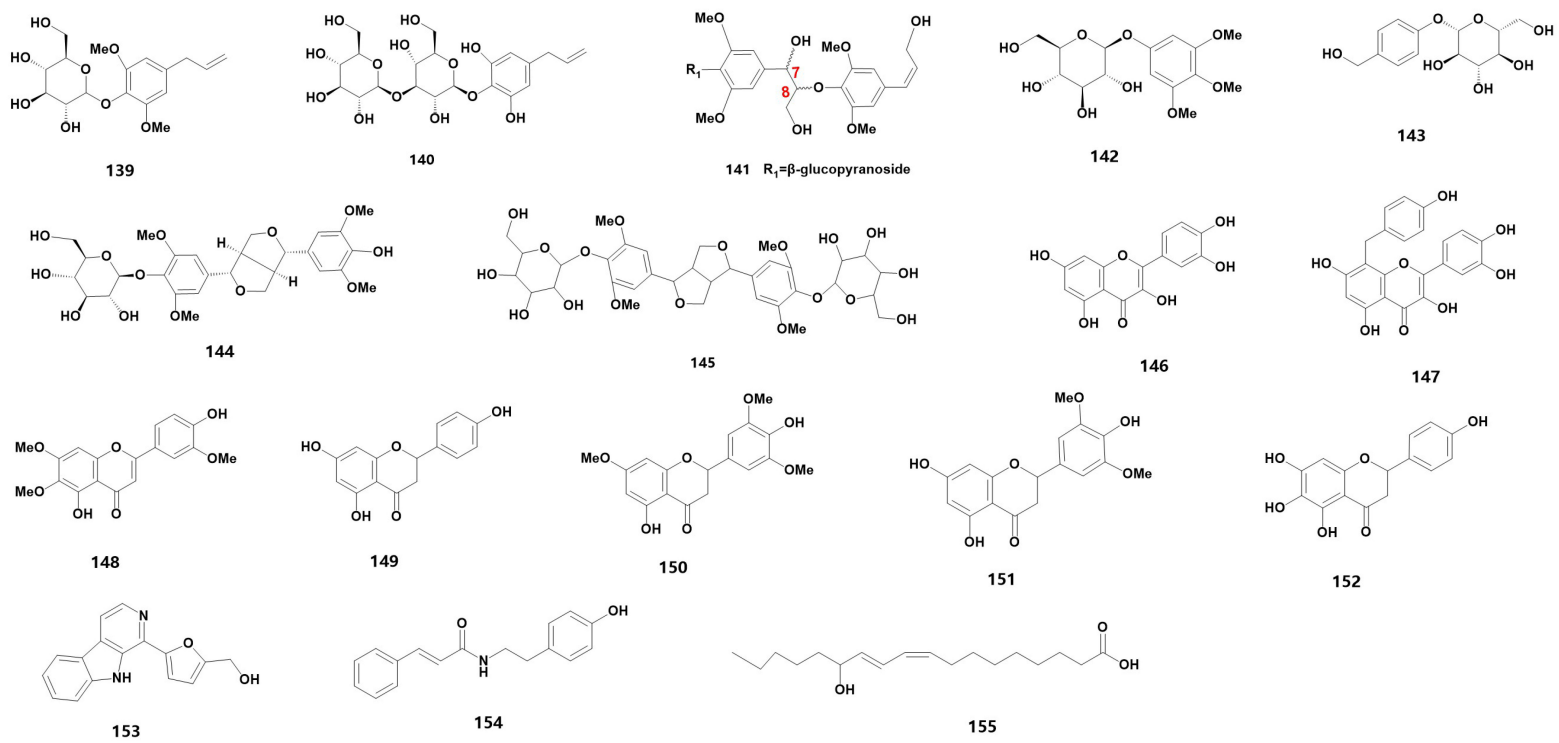
The chemical structure of other substances found in orchids.

Medicinal orchids encompass a wide range of species, each characterized by a diverse array of secondary metabolites. These metabolites can be further categorized into various material types. For instance, flavonoids can be further sub-classified into flavanones, flavonols and other categories. These flavonoids are widely distributed in medicinal orchid plants and are a primary source of plant pigments. In fact, 66 flavonoids have been detected in 24 orchid plants [23], while 34 different species of *Dendrobium* have been found to contain flavonoids [24], including *D. devonianum* [25], *D. officinale* [26,27,28], *D. fimbriatum* [29] and *D. huoshanense* [30]. In the family of Orchidaceae, many types of secondary metabolites still exist, and 54 species are summarized in Table 1 (102–155) (for their chemical structures, refer to Figure 4). Based on their structural characteristics, we have classified them into ethers (102–103), alcohols (104–109), aldehydes (110–114), coumarins (115–117), lignans (118–120), esters (121–129), phenolic acids (130–137), glycosides (138–145) and flavonoids (146–152).

**Table 1:** A total of 155 components were first identified from medicinal orchids in 2018–2023.

NO.	Compound	Plant Source	Reference
Alkaloids			
1	Dendrobine	C, P	[31,32]
2	N-isopentenyl-dendrobinium	C, P	[31,32]
3	N-isopentenyl-dendroxinium	C, P	[31,32]
4	Nobilonine	C, P	[31,32]
5	Dendramine	P	[32]
6	Mubironine A	P	[32]
7	Findlayine D	P	[32]
8 *	dendronboic acid	N	[8]
9	N-p-Cinnamoyl-tyramine	P	[32]
10 *	N-methoxylcarbonyldendrobine	N	[8]
11	Crepidine	P	[32]
12	Dendrocrepidine B	P	[32]
13	Dendrocrepine	P	[32]
14	Dendrocrepine C	P	[32]
15	Credidamine	P	[32]
16	Dendrocrepidine D	P	[32]
17	Homocrepidine B	P	[32]
18 *	Crepidtumines A	C	[10]
19 *	Crepidtumines B	C	[10]
20 *	Crepidatumines C	C	[9]
21 *	Crepidatumines D	C	[9]
22	ginsenine	BU, R	[33,34]
23	anocetochine	BU	[33]
24	Neoechinulin A	L	[35]
25	Indole-3-aldehyd	ST	[36]
26 #	acortatarin A	J	[37]
27	Huperzine A	BU	[33]
Phenanthrenes			
28 *	2,2′,2″,7,7′,7″-hexahydroxy-4,4′,4″-trimethoxy-[9,9′,9″,10,10′,10″]-hexahydro-1,8,1′,6″-triphenanthrene	ST	[12]
29	2,5-dihydroxy-4-methoxy-9,10-dihydrophenanthrene	PL, M	[20,38]
30	2,5,7-trihydroxy-4-methoxy-9,10-dihydrophenanthrene	B	[39]
31	coelonin	H	[40]
32	4,7-dimethoxy-9,10-dihydrophenanthren-2-ol	ST	[36]
33	2-methoxy-9,10-dihydrophenanthrene-4,5-diol	ST	[36]
34	4-methoxy-9,10-dihydrophenanthrene-1,2,7-triol	ST	[36]
35	1,4,7-trihydroxy-2-methoxy-9,10-dihydrophenanthrene	PL	[20]
36	calanhydroquinone C	PL	[20]
37 #	2,7-Dihydroxy-3,5-dimethoxy-9,10-dihydrophenanthrene	ST	[12]
38 #	2,3,7-Trihydroxy-4-methoxy-9,10-dihydrophenanthrene	ST	[12]
39 *	spiranthesphenanthrene A	SI	[13]
40 *	spiranthesphenanthrene B	SI	[13]
41 *	spiranthesphenanthrene C	SI	[13]
42 *	spiranthesphenanthrene D	SI	[13]
43 *	spiranthesphenanthrene E	SI	[13]
44 *	spiranthesphenanthrene F	SI	[13]
45	Dendrocandin P1	O	[41]
46	Dendrocandin P2	O	[41]
47 *	bletilore A	ST	[36]
48	chrysotoxol A	L	[35]
49	3,7-dihydroxy-2,4-dimethoxy-phenanthrene	PL	[20]
50	2,5-dihydroxy-4-methoxyphenanthrene	HA, M	[22,38]
51	2,5-dihydroxy-4,9-dimethoxyphenanthrene	HA	[22]
52 *	2-hydroxy-3,4,7-trimethoxyphenanthrene	O	[42]
53	2,4,8-trimethoxy phenanthrene-3,7-diol	N	[43]
54	5,7-dimethoxyphenanthrene-2,6-diol	ST	[36]
55	1,5-dimethoxyphenanthrene-2,7-diol	ST	[36]
56	7-hydroxy-2-methoxy-1,4-phenanthrenequinone	HA	[22]
57 *	Bulbocodioidins A (9R,9S)	BUL	[14]
58 *	Bulbocodioidins B (9R,9S)	BUL	[14]
59 *	Bulbocodioidins C (9R,9S)	BUL	[14]
60 *	Bulbocodioidins D (10S,10R)	BUL	[14]
61 *	2,3-dimethoxyl-7-hydroxyl-1,4-phenanthrenedione	F	[15]
62 *	2-methoxyl-3-methyl-7-hydroxyl-9,10-dihydro-1,4-phenanthrenedione	F	[15]
63	densiflorol B	N	[43]
64	cypripedin	N	[43]
65	3-hydroxymethyl-9-methoxy-2-(4′-hydroxy-3′, 5′-dimethoxyphenyl)-2,3,6,7-tetrahydrophenanthro [4, 3-b] furan-5, 11-diol	M	[38]
66 #	4,7,4′,7′-tetrahydroxy-2,2′-dimethoxy-1,1′-biphenanthrene	ST	[12]
Bibenzylates			
67 #	3-Hydroxy-5-methoxybibenzyl	ST	[12]
68	3,3′,5-trihydroxybibenzyl	L	[35]
69	batatasin III	L, M, B, H	[35,38,39,40]
70	3,4′-dihydroxy-5-methoxybibenzyl	H	[40]
71	3-hydroxy-4′,5-dimethoxybibenzyl	H	[40]
72	3-O-methylgigantol	H	[40]
73	gigantol	H	[40]
74	3,4-dihydroxy-4′,5-dimethoxybibenzyl	H	[40]
75 #	Moscatilin	PL, H	[20,40]
76	3,5,5′-trihydroxy-4′-methoxybibenzyl	ST	[36]
77	3-O-methyldihydropinosylvin	ST, M	[36,38]
78	Dihydropinosylvin	ST	[36]
79	3,5,5′-trihydroxybibenzyl	ST	[36]
80	3,5,4′-trihydroxybibenzyl	ST	[36]
81	4,3′,5′-trihydroxy-3-methoxybibenzyl	HA	[22]
82	4,3′-dihydroxy-3,5′-dimethoxybibenzyl	HA	[22]
83#	3′-hydroxy-3,4,4′,5-tetramethoxybibenzyl	PL	[20]
84	4,4′-dihydroxy-3,3′,5-trimethoxybibenzyl	M, HA	[22,38]
85	dendrosinen B	B	[39]
86	4,3′-dihydroxy-3,5-dimethoxybibenzyl	HE	[21]
87	4′, 5-dihydroxy-3, 3′-dimethoxybiphezyl	B	[39]
88	3, 3′-dihydroxy-4, 5-dimethoxybiphezyl	B	[39]
89 #	3-methylgigantol	PL	[20]
90 *	2-chloro-3,4′-dihydroxy-3′,5-dimethoxybibenzyl	PL	[20]
91	4,5-dihydroxy-3,3′,α-trimethoxybibenzyl	HE	[21]
92	dendrocandin A	H	[40]
93	(S)-3,4,α-trihydroxy-4′,5-dimethoxybibenzyl	H	[40]
94	densiflorol A	H	[40]
95	4,4′-dihydroxyl-3,5-dimethoxylbibenzyl	L	[35]
96	4,α-dihydroxy-3,5,3′-trimethoxybibenzyl	HE	[21]
97	dendrosinen D	B	[39]
98 *	3,4,α-trihydroxy-5,3′-dimethoxybibenzyl	HE	[21]
99	trigonopol B	L	[35]
100	4,4′-dihydroxy-3,5,3′-trimethoxybibenzyl	HE	[21]
101 *	3, α-dihydroxy-4,5,3′-trimethoxybibenzyl	HA	[22]
Other secondary metabolites			
102 #	p-hydroxybenzyl methyl ether	ST	[12]
103	p-Hydroxybenzyl ether	ST	[12]
104	p-hydroxybenzyl alcohol	ST	[12]
105	4-methoxy-phenylethanol	S	[44]
106	dihydroconiferyl alcohol	HU	[45]
107	anoectosterol	BU, R	[33,34]
108 #	(E) -4- (2-methoxyvinyl) benzene-1,2-diol	N	[43]
109	p-hydroxybenzyl alcohol	ST	[36]
110	3-hydroxybenzaldehyde	S	[44]
111	3,4-dihydroxy-5-methoxy benzaldehyde	HU	[45]
112 #	3,5-dihydroxy-4-hydroxy benzaldehyde	HU	[45]
113	4-hydroxy-3-methoxy benzaldehyde	HU	[45]
114	5-hydroxymethyl furfural	HU	[45]
115	coumarin	N	[43]
116 #	moellendorffiline	N	[43]
117 #	isopimpinellin	N	[43]
118	syringaresinol	L, HE	[21,35]
119	neoolivil	B	[39]
120	Matairesinol	S	[44]
121 #	methyl melilotate	M	[38]
122 #	ethyl melilotate	M	[38]
123 *	methyl 2-(acetyloxy) benzenepropanoate	M	[38]
124	Dihydroconiferyl dihydrop-hydroxycinnamate	B	[39]
125	eis-p-hydroxyl ethyl cinnamate	S	[44]
126	p-hydroxyphenylpropionic ethyl ester	S	[44]
127	methyl 3-(4-hydroxyphenyl) propionate	HU	[45]
128	(9Z,12Z)-methyl octadeca-9,12-dienoate	S	[44]
129	hexadecanoic acid 2,3-dihydroxypropyl ester	HE	[21]
130	p-hydroxyphenyl-propionic acid	B	[39]
131	p-hydroxybenzoic acid	HU	[45]
132	p-hydroxycinnamic ac	B	[39]
133	ferulic acid	B	[39]
134	caffeic acid	B	[39]
135	4-Hydroxy-2-methoxy-3,6-dimethylbenzoic acid	H	[40]
136 #	2-Hydroxy-4-methoxy-3,6-dimethylbenzoic acid	B	[39]
137	dihydroferulic acid	HU	[45]
138 #	4-O-β-D-glucopyranosyl coniferyl aldehyde	HU	[45]
139	4-allyl-2,6-dimethoxyphenyl glucopyranoside	HU	[45]
140 #	3,4,5-trihydroxyallylbenzene-3-O-β-D-glucopyranosyl-4-O-β-D-glucopyranoside	HU	[45]
141	(7S,8R)-syringylglycerol-8-O-4′-sinapyl ether 4-O-β-D-glucopyranoside	HU	[45]
142 #	3,4,5-trimethoxyphenol-1-O-β-D-glucopyranoside	HU	[45]
143	gastrodin	HU	[45]
144	(+)-syringaresinol-4-O-β-D-glucopyranoside	HU	[45]
145	Liriodendrin	HU	[45]
146	quercetin	BU	[33]
147	8-p-hydroxybenzyl quercetin	BU	[33]
148	5,4′-dihydroxy-6,7,3′-trimethoxyflavone	BU	[33]
149	naringenin	L, N, HU	[35,43,45]
150	5,4′-dihydroxy-7, 3′, 5′-trimethoxyflavanone	L	[35]
151	5,7, 4′-trihydroxy-3′, 5′-dimethoxyflavanone	L	[35]
152 #	carthamidin	H	[40]
153 #	periloyrine	HU	[45]
154	N-trans-cinnamoyltyramine	HE	[21]
155 #	(9Z,11E) -13-hydroxy-9,11-octadecadienoic acid	M	[38]

Notes: Compounds 1 to 27 are alkaloids, 28 to 66 are phenanthophylls, 67 to 101 are bibenzylls and 102 to 155 are classified as other compounds. * denotes newly identified compounds; **#** indicates the first isolation from this genus; the rest indicate the first isolation from this plant species. **C** refers to *D. crepidatum*, P refers to *D. pendulum*, N refers to *D. nobile*, L refers to *D. loddigesii*, PL refers to *D. plicatile*, M refers to *D. moschatum*, H refers to *D. heterocarpum*, B refers to *D. bellatulum*, O refers to *D. officinale*, HA refers to *D. hancockii*, F refers to *Flickingeria fimbriata*, HE refers to *D. hercoglossum*, HU refers to *D. huoshanense*, S refers to *D. sinense*, CH refers to *D. chrysotoxum*, W refers to *D. wardianum*, BU refers to *Anoectochilus burmannicus*, R refers to *A. roxburghii*, ST refers to *B. striata*, J refers to *Liparis japonica*, NE refers to *L. nervosa*, SI refers to *Spiranthes sinensis*, BUL refers to *P. bulbocobioides*, Y refers to *Pholidota yunnanensis*, SU refers to *Pecteilis susannae* and MI refers to *Phaius mishmensis*.

## 3. Pharmacological Activity and Mechanism of Secondary Metabolites of Medicinal Orchids

The diversity of secondary metabolite activity in medicinal orchids has been revealed through research, with a single component demonstrating a variety of effects. For example, DHP was initially confirmed to selectively inhibit HepG2 cells but has also been found to aid in bacteriostasis as an auxiliary component. Specifically, it can increase the sensitivity of strains such as *Staphylococcus aureus*, *Klebsiella pneumoniae* and *Candida albicans* to antibiotics, thereby reducing resistance and promoting antibacterial effects [46]. Additionally, DHP serves as an auxiliary treatment for neurological diseases [47]. The broad-spectrum biological activity of pharmaceutical secondary metabolites has undermined the “secondary metabolite waste” argument and provided strong support for their development and utilization.

### 3.1. Antibacterial Activity

Antibacterial active substances are often involved in the treatment process of larger wounds, used to prevent inflammation reactions caused by bacterial infections. Orchidaceae contains various active substances with bacteriostatic effects, such as phenanthrene, alkaloids and phenylpropylene. Yuan Jia et al. discovered that three phenanthine compounds, shanciol H, cebu sylene and pleionesin C, had a strong inhibitory effect on *Staphylococcus aureus*. The MIC_50_ value for the three compounds was between 74.965% and 53.494% at the level of 100 μmol/L, which was stronger than the positive drug penicillin G sodium, which had a value of 98.337% [48]. Additionally, the Filipino substances had moderate antibacterial properties against *S. aureus* and *Escherichia coli* [49]. Broad-spectrum bacteriostatic activity is a crucial factor in measuring the antibacterial activity of active substances. Dong et al. discovered that total alkaloids have an inhibitory effect on sixteen test bacteria, including twelve strains of *S. aureus*, Salmonella, Ginger blast bacteria and others, as well as four fungi. Nine test bacteria exhibited an inhibition circle diameter of over 15 mm for the total alkaloids at a concentration of 240 μg/mL, indicating that the bacteriostatic effect is broad spectrum and highly sensitive [50]. Secondary metabolites primarily act as bacteriostatics by interfering with the normal metabolism of strains, resulting in the death of pathogenic bacteria. For example, by disrupting the strain’s cell membrane and cell wall structure or interfering with mitochondrial (fungal) metabolism, destroys the original metabolism of pathogenic bacteria, inhibiting its metabolic reproduction [51,52].

### 3.2. Regulation of Free Radical Metabolism and Antioxidants

Free radicals are highly active substances, and their excessive accumulation can cause damage to the body. However, there are ways to balance the metabolism of free radicals, such as scavenging them, inhibiting their activity or activating the body’s antioxidant system. This helps slow down cell oxidation and prevent aging [53,54]. In response to this phenomenon, researchers have conducted extensive studies on the antioxidants found in medicinal orchids. For example, Yan Sha et al. discovered that phenanthrene secondary metabolites have efficient activity against free radical scavenging. At a concentration of 100μg/mL, they demonstrated a DHHP scavenging rate of 80% or more [55]. They also found that a particular category of polyphenols had higher antioxidant activity than that of vitamin C [35]. Additionally, bibenzyloids, first found in *D. heterocarpum*, have high activity in scavenging free radicals, with the IC_50_ of the ABTS radicals and DPPH radicals being 36.41 ± 1.99 and 62.05 ± 3.40 μmol/L, respectively [40]. White and mesoanthocyanidins also have antioxidant properties, with OH^−^ scavenging activity at 147.36 U/mL, a DPPH of 85.43% and O^2−^ at 18.02% [56]. These findings offer abundant sources for the development of antioxidants. However, some investigations suggest that these antioxidant compounds may also have negative effects on the human body. Therefore, it is important to carefully consider the dosage and application methods of these substances in clinical settings.

### 3.3. Biological Activity against Cancer Cells

In 2019, the World Health Organization estimated that cancer was the main or second leading cause of death among individuals under 70 years of age in 112 countries worldwide, with an astonishing 9.958 million deaths due to cancer in 2020 alone [57]. The identification of plant-specific biologically active substances has opened up new possibilities for cancer research, including camptothecin (from *Camptotheca*), paclitaxel (from yew bark), vincristine (from periwinkle), topotecan (a camptothecin derivative) and etoposide (a podophyllotoxylene derivative). These findings suggest that strengthening the development of plant active substances may aid in the fight against cancer. Medicinal orchids contain a variety of secondary metabolites with antitumor effects, which manifest as the inhibition of cancer cells, cytotoxicity, the induction of apoptosis and the inhibition of metastasis and spread (Table 2). The occurrence of cellular filamentous pseudopodia is a characteristic structure of cell movement, and the development of cancer cell metastasis or invasion can be determined by observing whether the pseudopodia extend outward. Phoyunnanin E can significantly decrease the number of pseudopodia in lung cancer H460 cells, as well as suppress cell migration by downregulating αv, β3 and migration regulatory proteins. Furthermore, it has a significant inhibitory effect on the migration of H292 and A549 human lung cancer cells [58]. Interestingly, derivatives of secondary metabolites derived from medicinal orchids have been found to exhibit anticancer activity. For instance, the bibenzyl derivative moscatilin has been shown to induce apoptosis in melanoma cells by regulating the p53 family, inhibiting the anti-apoptotic protein Bcl-2 and activating the pro-apoptotic protein Bax [59]. Additionally, moscatilin has been demonstrated to enhance the sensitivity of esophageal cancer cells to radiotherapy [60]. During cancer treatment, it is crucial to evaluate the potential harm that active substances may cause to a patient’s healthy body. Unfortunately, there is currently a dearth of research assessing this damage. Therefore, a comprehensive evaluation based on both the effectiveness of treatment and the possible harm caused should be considered an important criterion for measuring the anticancer activity of these substances. Given the abundance of species and secondary metabolites found in medicinal Orchidaceae plants, they are a highly promising subject to discover an optimal anti-cancer adjuvant agent.

### 3.4. Other Biological Functions and Potential Applications

In addition to the above, secondary metabolites of Orchidaceae also have a wide range of biological activities, such as the sedative, analgesic, antiepileptic, anticonvulsant, antiviral and other effects [71] of gastronomin, the potential therapeutic and preventive effects of alkaloids in Alzheimer’s disease [72], the strong anti-inflammatory and antioxidant activities of polyphenols [35] and so on. The vast treasure trove of medicinal orchids still needs research breakthroughs in many areas, such as in reducing pathogen resistance and improving therapeutic efficacy through pharmaceutical adjuvants. For example, the combination of mauranthin and doxorubicin hydrochloride uses folate-chitosan layering to wrap and target the two to the site of breast cancer cell action, and the inhibitory effect is increased by 30% compared with that of free drugs [73], enhancing the efficacy while reducing unnecessary losses.

### 3.5. Potential Activity in Organic Extracts

The diverse biological activity exhibited by medicinal orchid extracts is attributed to the presence of a wide range of secondary metabolites. For instance, the extract obtained from *D. wrinkled* leaves showed promising results in scavenging and inhibiting DPPH free radicals, as well as inhibiting the growth of cancer cells such as HeLa and U251 [74]. Similarly, the root extract of *Eulophia macrobulbon* was found to have potential in vitro anti-inflammatory and cancer cell toxicity effects, by reducing the production of pro-inflammatory factors, such as interleukin 6 and tumor necrosis factor α, and downregulating the expression of pro-inflammatory factor iNOS [75]. An identification analysis revealed the presence of secondary metabolites, such as total flavonoids, total phenols and phenanthrene in organic extracts [74,75]. Currently, natural products have not been widely used in the clinical treatment of obesity. However, it has been reported that the methanol extract of *D. heterocarpum* has the potential to regulate obesity by reducing the lipid storage capacity of 3T3-L1 pre-adipocytes, inhibiting the expression of key differentiation factors and ultimately affecting lipid storage and the differentiation of preadipocytes. The main substance responsible for this effect was identified as 3,4-dihydroxy-5,4′-dimethoxybibenzyl [76]. Interestingly, the efficacy of extract activity is also influenced by the type of solvent used. The biological activity of extracts obtained using different solvents can vary slightly, mainly due to differences in the affinity of the metabolites for the solvent.

## 4. Conventional Methods for Increasing the Yield of Secondary Metabolites of Medicinal Orchids

The traditional planting mode of medicinal orchids has several disadvantages that are not aligned with the requirements of social development. Their extended planting cycle, low yield and unstable quality are some of the issues that need to be addressed urgently. In this context, secondary metabolites play a pivotal role in determining the quality of medicinal orchids, and it is imperative to achieve a breakthrough in terms of their yield. The application of synthetic biology methods can replace or optimize the original indigenous planting mode to generate high-quality medicinal orchids that are rich in secondary metabolites. Some of the widely used methods include callus suspension cultures, the directed synthesis of exogenous substances, heterologous transformation and endophytic fungal co-cultures. In summary, the limitations of the traditional planting mode of medicinal orchids can be overcome by implementing innovative techniques that leverage the potential of secondary metabolites. The use of synthetic biology methods offers an opportunity to advance the synthesis and application of these valuable compounds, paving the way for high-quality development.

### 4.1. Plant Medium System

The traditional planting cycle is heavily influenced by natural conditions such as climate, season and habitat. This results in a long cycle that is difficult to control and is not efficient. To address this, ex vivo cultivation technology has been developed as an innovative solution. In vitro cultivation can be divided into three stages: the induction of seed germination, induction of callus formation and callus transformation into plants. The enhanced synthesis of secondary metabolites in plants, as a response to stress, can be achieved by providing suitable promoters and stressors during the cultivation process. Hormones, important signaling molecules that regulate substance anabolism, are also a major factor in the cultivation process. The treatment of *B. striata* and suspension cells with exogenous hormone auxin-naphthaleneacetic acid (NAA), auxin phytohormone indole-3-butyric acid (IBA) and cytokinin 6-benzylaminopurine 6-BA resulted in a significant increase in emodin methyl ether synthesis at a concentration of 1.0 mg/L [77]. Similarly, the treatment with 6-BA and auxin analogue 2,4-dichlorophenoxyacetic acid induced white and suspension cell cultures and led to a significant increase in the yield of p-hydroxybenzyl alcohol, militarine, dactylorhin A and coelonin [78]. It is worth noting that the stockpile of precursors is crucial in ensuring the synthesis of substances in the biosynthesis reaction. For instance, the addition of phenylalanine during periods (1–3) significantly increased the yield of moscatilin in *D. ovatum* callus [79]. In another example, the single-cell line culture of *D. huoshanense* with the addition of germanium, selenium, acid hydrolyzed casein and other substances improved the accumulation of alkaloids and flavonoids significantly [80,81]. Furthermore, the impact of the temperature variation between day and night on the production of coniferin and syringin has been researched [82]. Through the constant refinement of media components, precursors and triggering agents, this study holds significant importance as a reference for the industrial synthesis of secondary metabolites.

### 4.2. Biotransformation Pathways

The transformation of heterologous organisms is a crucial production method for the targeted synthesis of products through their biological properties. The tissue suspension culture technique is commonly employed in the biotransformation of secondary metabolites in medicinal orchids, such as the mutual conversion of gastrodin and p-hydroxybenzyl alcohol. For instance, in the suspension cell culture system of *Platycodon grandiflorum*, gastrodin added as an exogenous compound can be converted into p-hydroxybenzyl alcohol [83]. Similarly, the addition of p-hydroxybenzyl alcohol to the suspension cell culture of Leonurus enabled the total conversion rate of gastrodin to reach over 23% within 48 h [84]. Hairy roots are also widely used in the conversion and synthesis of secondary metabolites due to their reliable and quick growth. Recently, gastrodin was successfully transformed by ginseng hairy roots [85] and *Datura stramonium* hairy roots [86]. In the transformation experiment of *D. ovatum* hairy roots, it was found that enhancing the defense mechanism of plants can induce the synthesis of certain secondary metabolites [87]. Although this can enhance their defense mechanism, it also inhibits the formation of hairy roots.

### 4.3. Accumulation-Promoting Effect of Endophytic Fungi

**Figure 5:**
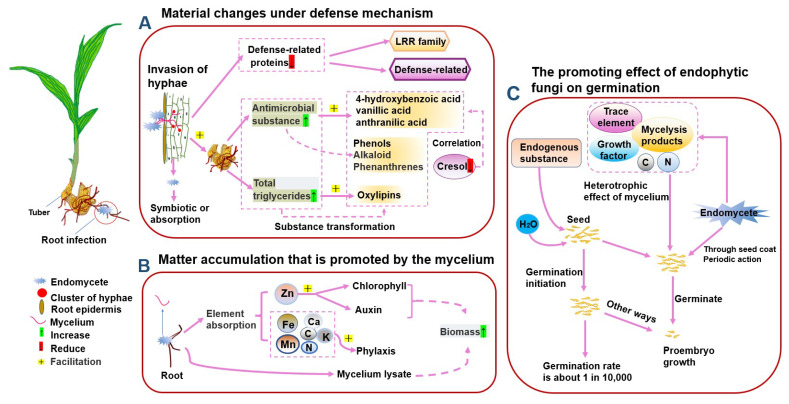
Synthetic metabolism of endophytic fungi on medicinal orchid plants. (**A**) Endophytic fungi boost the production of secondary metabolites through a biochemical reaction. This process involves reducing the defense protein content of the infection site while elevating the levels of antibacterial secondary metabolites and triglyceride content. (**B**) Endophytic fungi can provide a material basis for plant biosynthesis by enhancing the absorption of various ions in plant roots. Moreover, plants can directly absorb and utilize mycelium. (**C**) Orchid seeds contain low levels of essential nutrients, have limited access to internal substances and exhibit poor efficiency. In their natural state, the majority of orchid seeds undergo germination under the heterotrophic influence of endophytic fungi. This is particularly evident as endophytic fungi penetrate the initial embryo through mycelial action, providing diverse material foundations for seed germination.

Endophytic fungi have a significant positive impact on plant growth and reproduction, stress resistance and the regulation of metabolic pathways [88]. According to studies, they also promote the accumulation of secondary metabolites in medicinal orchids through signal recognition, transduction, the activation of enzymes and gene expression in secondary metabolic pathways [89]. Visual changes in the content of secondary metabolites can be detected through co-cultures with endophytic fungi. For instance, in a co-culture with *B. striata* callus, the content of p-hydroxybenzyl alcohol increased by two times on the 6th day compared to the control group, while the contents of dactylorhin A and 4-methoxy-9,10-dihydrophenanthrene-2,7-diol increased by two and one and a half times, respectively, on the 9th day. Moreover, it was observed that the content of a group of *B. striata* glycosides decreased by eight times at different times and with varying strains [90]. Another study found that spraying endophytic fungi on the surface of *D. officinale* seedlings resulted in an alkaloid content increase in the range of 10^−3^–10^−4^%, with the roots and stems showing significantly higher promotion effects compared to those of the leaves [91]. Studies have shown that the communication between endophytic fungi and medicinal orchids is mainly achieved through hyphal action [92]. During this process, the mycelial infection of endophytic fungi stimulates the defense mechanism of medicinal orchids, causing the specific synthesis of certain antibacterial substances, such as phenanthrenes, alkaloids and other compounds (Figure 5A). For example, when *Dactylorhiza* tubers were inoculated with fungi, the content of antimicrobial phenols and alkaloid precursors was detected, and the content of DHP significantly increased [93]. Additionally, the hyphae associated with the infection process are also an important heterotrophic source for orchid growth and can provide the material basis for their biomass synthesis (Figure 5B). Interestingly, the heterotrophic effects of hyphae can also promote the germination of medicinal orchid seeds [94] (Figure 5C).

### 4.4. Synthesis of Secondary Metabolites under Environmental Stress

The environment plays a crucial role in supporting the growth and development of plants by providing the necessary conditions and essential factors for their metabolism. In biology, environmental stressors can encompass changes in various environmental factors, such as light, temperature, salinity, humidity and others. Among them, light is an important factor affecting the material transformation of plants. After irradiating the protocorm-like bodies (PLBs) of *Dendrobium* hybrid orchids with light-emitting diodes, it was discovered that this technique could enhance the synthesis of flavonoids and polyphenols in PBLs [95]. However, excessively bright or weak light can cause plants to experience light inhibition. Under high-intensity light stress, *Phalaenopsis aphrodite* can enhance their tolerance to high-intensity light through blue light treatment, as evidenced by the increased levels of the anthocyanin and chlorophyll content [96]. In *D. officinale*, it has been observed that high-intensity light has the ability to activate the gene DoHY5. This activation in turn triggers a DoHY5-dependent signaling pathway that promotes the synthesis of flavonoids and polysaccharides [97]. Under light stress, the synthesis of flavonoids is regulated by chlorophyll, the main pigment. During the cultivation of *D*. *officinale*, it has been observed that high levels of selenium can hinder the synthesis of chlorophyll [98]. Additionally, the synthesis of flavonoids and alkaloids is impeded when the plant is subjected to low levels of phosphorus stress [99]. Under such stress, photosynthesis is also an important factor that affects substance synthesis. Additionally, plants also demonstrate a decrease in antioxidant activity, indicating a reduction in the content of reducing substances or a decline in the expression of reducing enzymes. Under ultraviolet (UV-B) radiation, the levels of alkaloids and flavonoids in plants increased significantly, accompanied by a decrease in chlorophyll and even a yellowing of the leaves [100,101]. Flavonoids and alkaloids are defensive compounds that help plants resist stress. For example, flavonoids are better at absorbing ultraviolet light. Environmental stressors have a two-way effect on the synthesis of secondary metabolites. Finding and fully exploiting a balance point can greatly improve the biosynthesis of secondary metabolites.

## 5. Prospectives

The secondary metabolites present in medicinal orchids exhibit a diverse range of biological activities. An in-depth analysis and comprehensive exploration of their active secondary metabolites can aid in clinical treatment and drug development in healthcare. This paper provides an overview of the various types and biological functions of the secondary metabolites found in medicinal orchids, alongside a discussion on conventional methods used to improve their yield. This lays the foundation for the application of orchids’ active substances at multiple levels. However, the application of medicinal orchid plants has not been precise, and several pharmacological mechanisms remain unclear. Additionally, the synthesis pathways of the vast majority of their active substances have not been deciphered, resulting in an inability to achieve the targeted synthesis and large-scale production of the active substances, hindering the promotion and modern development of orchids in traditional Chinese medicine.

### 5.1. Combined Application of Active Secondary Metabolites

Traditional CHM prioritizes the compatibility of medicinal materials and multi-course treatments as its core, while modern medicine focuses on precision, efficiency, quantification and systematization. In this context, the integration of multiple active substances has become a development trend. Under the combined action of substances, the ideal therapeutic effect is mainly achieved through the complementarity of their activities, which slows down toxic and side effects while enhancing their overall availability. For instance, the combination of calycosin and adriamycin mentioned earlier has shown that together they are greater than the sum of their parts, achieving a remarkable synergy. Both herbal treatments and combinations of drugs face a common problem. Combined treatment often refers to a precise combination to ensure compatibility. In the present herbal industry, there lacks a unified evaluation system for assessing the quality of medicinal materials. Consequently, the established compatibility standards exhibit substantial regional and subjective variations. This situation deviates from the requirements of modern medicine. Therefore, traditional Chinese medicine has great development potential to extract active substances for research in combined treatments. In the future, the combination of active substances could become a driving force to promote the development of herbal compatibility therapy.

### 5.2. Application Optimization of Active Secondary Metabolites

With the continual discovery of new active substances and analyses of existing active substances, highly specific active substances will not become scarce anytime soon. To a certain extent, the activity of secondary metabolites is closely linked to their method of use. For instance, protein drugs are unsuitable for oral administration. This revelation highlights the importance of controlling physical and chemical properties, as well as physiological properties, when studying the activity of substances. For example, the structural characteristics of certain substances may hinder their bioavailability, and employing microbial transformations to reduce hydrophobicity and minimize toxic side effects is of significant practical importance. Therefore, finding a suitable application system for the corresponding active substance and accurately grasping its physical and chemical properties are important factors to consider when realizing its clinical application and value.

### 5.3. Modern Reproduction and Breeding of Medicinal Orchids

Modern cultivation technology is highly diversified, with transgenic technology being widely used in genetic breeding. In this context, establishing an original medicinal orchid gene database is essential. It not only protects original materials but also optimizes the breeding process. In China, Chinese medicine is a common health food. Establishing an efficient cultivation system for Chinese medicinal materials, such as suspension cultivation, holds immense social importance as it enables the entry of valuable medicinal resources into the population. This breeding technology will effectively address the continuous depletion of wild medicinal orchid resources.

With the continuous iteration and development of multi-omics biological analysis technologies, high-precision instruments, new materials and research ideas, there will be a clearer understanding of the active substances of medicinal orchid plants regarding their gene regulation, material metabolism, pharmacological mechanism and clinical application. This enhanced understanding will inject fresh vitality into traditional Chinese medicine, increasing the transparency of CHM and propelling the development of modern Chinese medicine to even higher levels.

## Figures and Tables

**Table 2:** Anticancer active substances and their functions.

Plant Source	Active Substance	Activities and Indicators	Cell Model	Reference
CH	Erianin	Inhibits the proliferation of cancer cells, promotes G2/M phase arrest and induces apoptosis and has a potential role in ferroptosis and the inhibition of the migration of lung cancer cells.	Lung cancer cell lines H1299 and H460	[61]
N	Crepidatin(1), Chrysotobibenzyl (2), 4,4′-Dihydroxy-3,3′,5-trimethoxydibenzyl (3)	Inhibit cancer cell proliferation. The IC_50_ values of (1)–(3) were 74.30 ± 0.98 μmol/L, 56.60 ± 0.92 μmol/L and 8.68 ± 0.95 μmol/L, respectively.	Hepatocellular carcinoma cell line FHCC-98	[62]
HA	Phoyunnanin E	Destroys the original morphology of H460 nucleus, promotes apoptosis and necrosis, and inhibits the migration of H460, H292 and A549 human lung cancer cells.	Human lung cancer H460, H292, A549 cells	[58]
Y	4,5-Dihydroxy-2-methoxy-9,10-dihydrophenanthrene (1),4,7-Dihydroxy-2-methoxy-9,10-dihydrophenanthrene (2)	Inhibition of the value added. The IC_50_ values of (1–2) were 25.5 and 29.1 μmol/L, respectively. (1) It can block HepG2 cells in the G2/M phase and induce apoptosis.	Human hepatoma cell line HepG2	[63]
W	4-hydroxy-3-methoxy cinnamaldehyde, 3,7-Dimethoxy-5-hydroxy-1,4-phenanthraquinone	Cancer cells are widely toxic. The IC_50_ values of the two compounds were 0.908–8.84 mol/L, and the inhibitory effect on tumor cells was stronger than that of the positive drug cisplatin.	Human promyelocytic leukemia cell line HL-60, human non-small cell lung cancer cell line A-549, human colon cancer cell line SW480, human SMMC-7721, human breast cancer cell MCF-7	[64]
SU	2,5-dihydroxy-4,9-dimethoxyphenanthrene (1), 4-methoxyphenanthrene-2,7-diol (2), 2,3-dimethoxy-1,4-phenanthrenequinone (3), 3,5,7-trihydroxyflavone (4)	They inhibited the growth of cancer cells (TS = 1.1–2.7) and had a certain tumor-specific cytotoxicity. Activity (1) > (4) > (2) > (3)	Human oral squamous cell carcinoma cells (HSC-2, HSC-3, HSC-4, Ca9-22), human promyelocytic leukemia cells HL-60	[65]
CH	Erianin, Chrysotoxine, Chysotoxene, confusarin	Proliferation inhibition (at an IC_50_ concentration of 50%, the inhibition rate was 0.0065,5.43,0.32,46.15 g/mL) at 72 h, with the increase in the compound concentration, the inhibitory effect was enhanced.	Chronic myeloid leukemia cell line K562	[66]
MI	phaitanthrin A(1),tryptanthrine(2)	Strong cytotoxicity. (1) The IC_50_ values for the two cancer cells were 33.8 and 27.0 μmol/L, respectively. (2) The IC_50_ values were 11.1 and 9.0 μmol/L, respectively.	Breast cancer cell line MCF-7, lung cancer cell line NCI-H460	[67]
E	Gastrodin	Led to the dose-dependent inhibition of cell proliferation and concentration-dependent induction of glioma cell apoptosis. The expression of p62 protein was significantly upregulated, the expression of LC3-II (or LC3-I) was decreased and Beclin1 protein was downregulated.	Glioma cell T98G	[68]
ST	7-hydroxy-2-methoxy-phenanthrene-3,4-dion,3′,7′,7-trihydroxy-2,2′,4′-trimethoxy-[1,8′-biphenanthrene]-3,4-dione	The two compounds have strong cytotoxicity. The IC_50_ values were close to that of the positive drug cisplatin. It can effectively induce the arrest of A549 cells in the G0/G1 phase, increase the production of reactive oxygen species (ROS) and promote the apoptosis of cancer cells.	Human lung cancer alveolar basal epithelial cells A549, human breast cancer cells MCF-7, human colon cancer cells HT-29	[69]
NE	2,2’,7’-trihydroxy-4,7,5’,6’-tetramethoxy-1,1’-biphenanthrene (1),2,7,2′-trihy- droxy-4,4′,7′-trimethoxy-1,1′-biphenanthrene (2),2,2′-dihydroxy-4,4′,7,7′-tetramethoxy-1,1′-biphenanthrene (3)	Strong cytotoxicity. (1–3) The IC_50_ values of HGC-27 were 8.21–9.95 μmol/L; (1) (3) The IC_50_ of HT-29 was 8.53–9.27 μmol/L.	Human colon cancer cell line HT-29, human gastric cancer cell line HGC-27	[70]
PL	2-chloro-3,4′-dihydroxy-3′,5-dimethoxybibenzyl	Strong cytotoxicity. The IC_50_ values for cancer cells were 3.41, 3.02 and 2.80 M, respectively.	MDA-MB231, HepG2 and A549 cells	[20]
N	densiflorol B (1), cypripedin (2), moscatin (3)	Significantly inhibit the proliferation of cancer cells. The IC_50_ of (1–3) were 2.99, 5.01 and 72.68 μmol/L, respectively.	MCF-7 breast cancer cells	[43]
O	orchinol	Significant killing effect on model cancer cells. The IC_50_ values were 11.96 and 8.92 μM, respectively.	Human promyelocytic leukemia cell lines HI-60 and THP-1 cells	[41]
SI	spiranthesphenanthrene A	The cytotoxicity was higher than that of cisplatin (IC_50_ = 19.0 ± 7.3 μM). By significantly increasing the level of E-cadherin and reducing the levels of vimentin, N-cadherin and Snail, the migration of cancer cells was significantly inhibited.	B16–F10 cancer cells	[13]

Notes: N refers to *D. nobile*, PL refers to *D. plicatile*, O refers to *D. officinale*, HA refers to *D. hancockii*, CH refers to *D. chrysotoxum*, W refers to *D. wardianum*, ST refers to *B. striata*, NE refers to *L. nervosa*, SI refers to *S. sinensis*, Y refers to *P. yunnanensis*, SU refers to *P. susannae*, MI refers to *P. mishmensis* and E refers to *G. elata*.
